# Does CD1a Expression Influence T Cell Function in Patients With Langerhans Cell Histiocytosis?

**DOI:** 10.3389/fimmu.2021.773598

**Published:** 2021-12-10

**Authors:** Jenée Mitchell, George Kannourakis

**Affiliations:** ^1^ Fiona Elsey Cancer Research Institute, Ballarat, VIC, Australia; ^2^ Federation University Australia, Ballarat, VIC, Australia

**Keywords:** CD1a-restricted T cells, CD1a, T cells, LCH cells, Langerhans cell histiocytosis (LCH)

## Abstract

Langerhans cell histiocytosis lesions are characterized by CD1a+ myeloid lineage LCH cells and an inflammatory infiltrate of cytokines and immune cells, including T cells. T cells that recognize CD1a may be implicated in the pathology of many disease states including cancer and autoimmunity but have not been studied in the context of LCH despite the expression of CD1a by LCH cells. In this perspective article, we discuss the expression of CD1a by LCH cells, and we explore the potential for T cells that recognize CD1a to be involved in LCH pathogenesis.

## Introduction

Langerhans cell histiocytosis (LCH) is a rare disorder characterized by an accumulation of myeloid lineage ‘LCH’ cells that co-express CD1a and CD207 (1, 2). Alongside an inflammatory infiltrate of cytokines and immune cells, the LCH cells form single or multiple lesions in various organs, with common sites including osseous and cutaneous tissue (3). LCH can affect all ages, although it is most commonly detected in infants and children. Relapse, long-term sequelae, and mortality effects many patients, therefore a greater understanding of the mechanisms that cause disease and better treatment options are required. The typical inflammatory environment within lesions suggests that there are many potential targets for new immune based treatments worthy of investigating and given their leading role in cell mediated immunity, T cells are key contenders.

LCH cells commonly harbor RAS/RAF/MEK/ERK pathway mutations, the most frequent of which is BRAF V600E (4–10). LCH cells are suggested mostly to have myeloid cell origin and similarities with antigen presenting cell lineages (11–15), although some lesions were recently found to arise upstream of these lineages in multipotent progenitor cells (16). Given the comparability with antigen presenting cell lineages, LCH cells likely have the ability to interact directly with T cells through contact-dependent and -independent manners. Additionally, BRAF V600 mutations have been detected in lymphocytes in a few instances (16, 17). T cells thus may be critical to LCH pathogenesis and indeed this has already been suggested for various reasons, such as the presence of cytokines suggestive of T cell activation (18, 19), and an enrichment of Foxp3+ regulatory T cells (Tregs), which are known for their immunosuppressive properties, within lesions (7-30% of total T cells) and blood from patients with active LCH (20, 21). Furthermore, cytotoxic T cell subsets have been highlighted in the LCH literature of late. These include CD8+CD56+ T cells and mucosal associated invariant T (MAIT) cells, which are both reduced in the peripheral blood of patients with LCH (median <1% of total T cells for each subset) (21, 22).

It is unsurprising that T cells would be involved in LCH pathogenesis given that LCH cells have been described as differentiating from monocytes (13, 14) or CD1c+ blood derived dendritic cells (DCs) (11, 12), thus probably capable of presenting antigen to T cells. LCH cells were reported to have mostly internalized major histocompatibility complex (MHC) II (23, 24), which limits the likelihood of peptide antigen presentation by LCH cells to CD4+ T cells within lesions. In contrast to their expression of MHC II, LCH cells almost ubiquitously express CD1a on their surface, and CD1a is known to house a range of endogenous and exogenous lipids, some of which can prime T cells for specific immune responses (25–31).

Group 1 CD1-restricted T cells collectively, which include T cells that recognize CD1a, have been suggested as prime candidates of immunotherapeutic potential by experts in the field (32, 33). This concept arose from both primary studies, and because of parallels emerging amongst these subsets and other more well-established unconventional T cell subsets such as type I natural killer T (NKT) cells and MAIT cells, that interact with non-polymorphic MHC-like molecules, and are able to bias immune responses (32–37). Examples of primary studies are those which demonstrated CD1-restricted T cell recognition of tumor derived lipids and cytotoxicity towards tumor cells (38, 39).

Compared to MAIT cells and type I NKT cells, to date little is known about T cells that recognize CD1a. We do know, however, that CD1a expression is altered in several disease models (40), including aberrant expression in cancers such as hairy cell leukemia (41) and some T lymphoblastic lymphomas (42). Aside from its association with LCH, CD1a is also linked with autoimmune skin diseases such as atopic eczema (43, 44) and psoriasis (45), thus T cells that recognize CD1a may be implicated in the pathology of many disease states, including cancer and autoimmunity. T cells that recognize CD1a have been detected in the skin and blood from healthy individuals using a variety of lipids in the form of endogenous ligands and exogenous antigens (25–27, 46). T cells that recognize CD1a have not been studied in the context of LCH but given the expression of CD1a by LCH cells, they may be involved in LCH pathogenesis. In this article the expression of CD1a in LCH and the plausible relationship between CD1a and T cells in LCH will be discussed.

## CD1a Molecules

**Figure 1:**
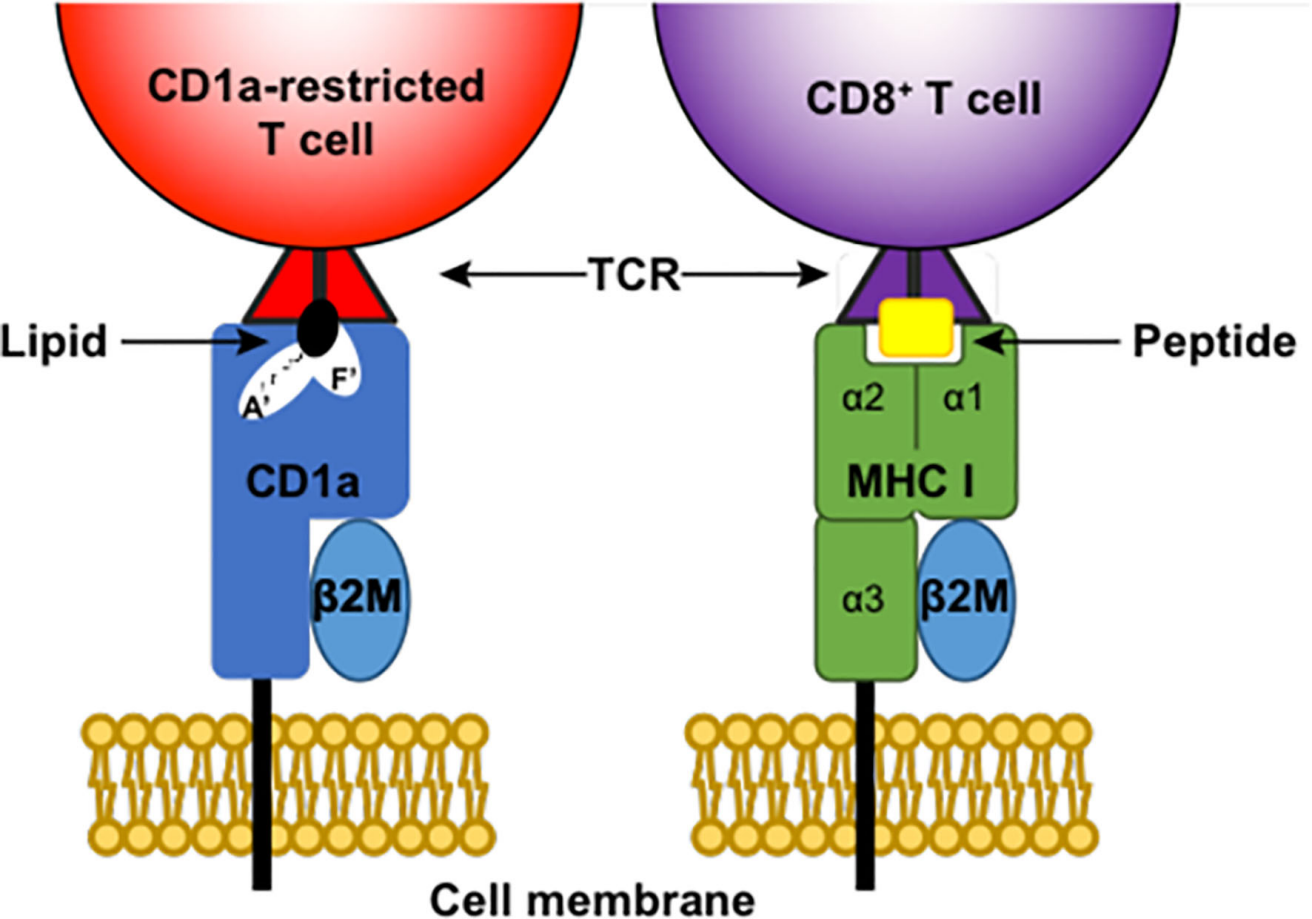
Antigen presentation by CD1a and MHC I. CD1a and MHC (Major histocompatibility complex) I are both membrane-bound complexes that can present antigen to T cells *via* the T cell receptor (TCR). Both complexes associate with beta2 microglobulin (β2M). MHC I presents peptide antigen to CD8+ T cells, while CD1a complexes can house lipids in both the A’ pocket and F’ pocket of their large hydrophobic grove and present these lipids to CD1a-restricted T cells.

CD1 molecules differ to classical MHC molecules because they display a large hydrophobic groove (47) that can present lipids and glycolipids to T cells (48) rather than the typical MHC format of peptide presentation (**Figure 1**). The CD1 groove consists of two large pockets, namely the A’ pocket and the F’ pocket, which can accommodate lipids with either short fatty acid tails combined with spacer lipids, or lipids with longer fatty acid tails (49–51) (**Figure 1**). CD1 complexes have some structural similarities to MHC I, for example both CD1 and MHC I molecules are associated with beta2 microglobulin (**Figure 1**). CD1 molecules may also share functional features with MHC II, as the invariant chain chaperone (CD74) that is involved in assembly and trafficking of MHC II is possibly associated with CD1 molecules and their trafficking (52). A key feature of CD1 molecules is their non-polymorphism, which means that targeting these molecules and/or the T cells they interact with may have the benefit of being a one-size-fits-all treatment approach in contrast to targeting highly polymorphic MHC molecules.

## CD1a Expression in LCH Lesions

CD1a is one of the diagnostic indicators for, and a hallmark of LCH. Expressed on the surface of LCH cells, the exact role of CD1a in LCH is unknown, but given its ubiquity, it could be a principal factor in LCH pathogenesis. An exciting CD1a-targeting treatment prospect highlighted in recent literature is CD1a chimeric antigen receptor T cell therapy (53). This technique has displayed promising preclinical results in the treatment of primary CD1a-expressing cortical T cell acute lymphoblastic leukemia cells (53). The clinical results may have implications for the treatment of LCH too, given the authors suggest its potential use in LCH due to the expression of CD1a by LCH cells and the low risk of off-target toxicity (53).

It is not currently understood why CD1a expression occurs on LCH cells and what the consequences of this expression are. Whilst it may be cytokine-induced, there are many studies that suggest varied conditions affect the expression of CD1a by antigen processing and presenting cells (54–56). CD1a can be upregulated by monocytes in the presence of stimuli such as phospholipids (phosphatidylcholine) or lectins (phytohemagglutinin) (54). In contrast, the environmental pollutants, polycyclic aromatic hydrocarbons (PAHs), were shown to inhibit CD1a expression in monocytes in the presence of dendritic cell (DC) differentiation cytokines, granulocyte-macrophage colony-stimulating factor (GM-CSF) and interleukin-4 (IL-4), but these pollutants did not alter CD1a expression by mature DCs (55). PAHs also impaired monocyte-derived DC maturation and consequently these cells poorly stimulated T cells in mixed leukocyte reactions (55). This impairment is akin to that observed in LCH cells, although the addition of CD40 ligand increased the allostimulatory activity of LCH cells (23). It would be compelling to know what effect this correction had on CD1a expression levels in LCH. There are abundant other factors, including lower than optimal temperature conditions (57), that can impact the regulation of CD1a. Whether one or more of these factors or an unknown mechanism is intrinsic to regulating CD1a expression in LCH lesions remains to be elucidated. Irrespective of how this occurs, CD1a expression in the peripheral blood by LCH-like cells and in lesions by T cells and LCH cells is likely important. The CD1a complex can bind a variety of lipids, some of which can prime T cells for specific immune responses (28), thus there are conceivable downstream signaling responses occurring as a result of CD1a expression. These T cells that can recognize and/or respond to CD1a-lipid complexes will be referred to herein as CD1a-restricted T cells.

## CD1a-Restricted T Cells and Their Potential Impact in LCH

CD1a-restricted T cells play a vital role in skin immunity (58), however the circulatory presence of these cells also infers a probable role for them more systemically in other host tissues (25, 28). This highly specialized subset of T cells are prime candidates for potentially influencing inflammation in LCH. There are a variety of known lipids that CD1a can bind, some of which have been shown to activate T cells or at least be recognized by the T cell receptor expressed by CD1a-restricted T cells. CD1a can bind several naturally occurring endogenous ligands such as squalene, wax esters and triacylglycerides commonly found in the skin oil (28). CD1a is also known to bind sphingomyelin, which is found in cell membranes and myelin sheath (31). Kasmar and colleagues (27) found that CD1a can bind the mycobacterial lipopeptide antigen dideoxymycobactin (DDM). The study found CD1a-DDM tetramer+ T cells in the peripheral blood of tuberculosis patients and showed that DDM is capable of binding to a recombinant TCR following incubation with CD1a (27). Additionally, the self-lipid galactosylceramide 3-sulfate (sulfatide) can stimulate multiple categories of CD1-restricted T cells including CD1a-restricted T cells (59). Phosphatidylcholine can activate CD1a-restricted T cells (60) and T cells were also reported to recognize CD1a-bound lysophosphatidylcholine (LPC) (31), both of which can be microbial- or self-derived. Other studies demonstrated that bee-venom and house dust mite derived phospholipase can generate small neo-antigens, for example free fatty acids and lysophospholipids from common phosphodiacylglycerides, which can in turn activate T cells *via* CD1a (29, 61). Plant-derived urushiol was also shown to trigger CD1a-dependent inflammation by CD4+ T helper (Th) cells (58). There are perhaps still many unknown lipids, either endogenous or exogenous that can bind CD1a and command a T cell response.

Little is known about the characteristic immune response of CD1a-restricted T cells. T cells that recognize CD1a can express the skin homing C-C chemokine receptors CCR4, CCR6 and CCR10 and produce IL-22, interferon gamma, TNF, GM-CSF and IL-13 (25, 28, 61). The production of these cytokines upon recognition of lipids presented by CD1a indicates that CD1a-restricted T cells can produce a Th1, Th2 or Th22 response (25–27, 61). Additionally, a preliminary study by the de Jong group [unpublished data reported by de Jong et al (62)] found CD1a-autoreactive T cells have clustered transcriptionally with Tregs. These studies suggest that alike other unconventional T cell lineages, CD1a-restricted T cells can bias an immune response. Moreover, studies have shown that TCRs can directly bind CD1a when permissive lipids such as phosphatidylcholine and other endogenous ligands form a complex with CD1a but do not protrude from the groove (20), which suggests that TCRs are able to directly recognize CD1a molecules. This implies that in addition to the type of lipid within the environment, the expression levels of CD1a in a particular environment may be a crucial factor in immunogenicity irrespective of the specific lipids being housed. It is possible that CD1a-restricted T cells are capable of additional immune responses, but our knowledge of their functions is currently limited.

Frequencies of antigen specific unconventional T cell subsets are commonly higher than frequencies of peptide antigen-specific conventional T cells. CD1a autoreactive T cell frequencies are similar to type I NKT cells and somewhat lower than MAIT cells. Recent research demonstrated that 1% of skin T cells produced cytokines in a CD1a-dependent manner without the addition of antigens (46). If CD1a-restricted T cells are activated by LCH cells they might proliferate within lesions and/or display an activated phenotype and produce cytokines that contribute to the inflammatory environment. Given the expression of LCH-like precursor cells found in the blood (63, 64) we could conceivably see changes due to CD1a-restricted T cells in the peripheral blood from LCH patients too. If CD1a-restricted T cells are present, activated or increased in frequency within LCH lesions, this may be a direct result of CD1a expression by LCH cells, and could even suggest antigenicity within lesions. Given we have previously detected a reduction in CD8+CD56+ T cells in patients with active LCH and highlighted a potential role for cytotoxic T cells in LCH pathogenesis (21) it would not be out of the question to consider that pro-inflammatory cytokine producing CD1a-autoreactive cells could have a potential role in the treatment of LCH. CD1a tetramers, which have only become available recently, may be useful to identify whether CD1a-restricted T cells are present in lesions or blood of patients with LCH and further indicate if they are involved in LCH pathogenesis. A caveat to testing LCH tissues with CD1a tetramers is that we don’t know how many lipids there are that are not currently known to bind CD1a, but potentially could. Using unloaded tetramers (46, 65) may resolve this in the context of investigating CD1a autoreactivity.

Interestingly, bee-venom phospholipase A2, which can trigger CD1a-dependent T cell activation, can also induce Foxp3+ Treg expansion through the modulation of apoptosis (66). This indicates that a mechanism that is responsible for CD1a-restricted T cell activation can also induce a Treg response, such as that seen in those with LCH. Whether this is true for LCH is worthy of investigation given the expression of both CD1a and an enrichment of Tregs in LCH lesions, and considering the transcriptional clustering of CD1a-autoreactive T cells with Tregs. Of note Tregs in LCH lesions express CD56 (21). This marker, known as neural cell adhesion molecule, is not usually expressed by Tregs, but is often seen on unconventional T cell subsets (67–69). These CD56-expressing Tregs could conceivably originate from an unconventional T cell lineage, although studies are needed to determine their origin. It is unknown whether CD1a-restricted T cells are present in LCH lesions, but given the expression of CD1a by LCH cells it is possible that there could be direct interactions between LCH cells and CD1a-restricted T cells, and indeed there are already established interactions between LCH cells and Tregs within lesions (20). Research into the associations between CD1a expression, CD1a-restricted T cells and Tregs in LCH lesions are certainly warranted as there is potential to unlock a key mechanism in LCH.

## Concluding Remarks

In this review, we have discussed what is currently known about CD1a expression in LCH and we highlighted the possible impacts that CD1a expression in lesions might have on T cells. Whilst there is prospective opportunity to target these cells to develop new immunotherapies, first we must better understand their precise role in the immune system and LCH alike. As our knowledge of this new subset of unconventional T cells increases, and reagents to facilitate studies become available, investigating the role of CD1a-restricted T cells in LCH is essential to directly addressing whether CD1a expression by LCH cells influences T cell function in patients with LCH.

## Data Availability Statement

The original contributions presented in the study are included in the article/supplementary material. Further inquiries can be directed to the corresponding author.

## Author Contributions

JM prepared the figure and wrote the manuscript. GK critically reviewed the manuscript. All authors contributed to the article and approved the submitted version.

## Conflict of Interest

The authors declare that the research was conducted in the absence of any commercial or financial relationships that could be construed as a potential conflict of interest.

## Publisher’s Note

All claims expressed in this article are solely those of the authors and do not necessarily represent those of their affiliated organizations, or those of the publisher, the editors and the reviewers. Any product that may be evaluated in this article, or claim that may be made by its manufacturer, is not guaranteed or endorsed by the publisher.
